# Tackling neighborhood health inequalities in the UK through cross-sector collaborations and community engagement: thematic synthesis of focus group and questionnaire data

**DOI:** 10.3389/fpubh.2026.1832109

**Published:** 2026-05-29

**Authors:** Linda J. M. Thomson, Rabya Mughal, Helen J. Chatterjee

**Affiliations:** 1 Department of Biosciences, University College London, London, United Kingdom; 2 Department of Arts and Sciences, University College London, London, United Kingdom

**Keywords:** community assets, community engagement, cross-sector collaborations, health inequalities, qualitative data, thematic analysis, thematic synthesis

## Abstract

**Background:**

Community engagement has become an increasingly common approach to addressing health inequalities. A three-phase UK-wide interdisciplinary, multicentered program of research, “Mobilizing Community Assets to Tackle Health Inequalities,” was established with the objective of utilizing local, cultural, and natural assets to address neighborhood health inequalities. Theprogram aimed to determine core components of cross-sector collaborations conducted by research teams comprising academic and non-academic, health and social care, local authority, voluntary and community partners.

**Methods:**

A thematic synthesis was performed on qualitative data comprising two peer-reviewed articles reporting focus group findings and two grey-literature reports synthesizing questionnaire data from the first two phases of the program. Themes occurring at least twice across these studies were aggregated into 15 descriptive themes, from which six overarching analytical themes were derived.

**Results:**

Relationship-building, multidisciplinary working, and training and capacity building ensured effective cross-sector collaborations. Additional enablers were integrated-care approaches, innovative creative-health solutions, and strategies for sustaining, replicating, and scaling up longer term projects. Creative and participatory research methods further strengthened evidence collection. Less consistently identified, though valuable in specific contexts, were interventions focused on raising awareness of poverty and deprivation, addressing social determinants of health, promoting health behaviors, empowering communities to make health-related decisions, supporting community-led solutions, and volunteering.

**Conclusion:**

Findings underscore the necessity of building credibility and trust, understanding cultural relevance to address non-participation by underserved communities, and enabling community empowerment. The research provides a more complete knowledge of community engagement and cross-sector collaborations than might have been derived from a single study.

## 1 Introduction

Community engagement has become an increasingly common approach to addressing health inequalities (1), that moves beyond historic models in which interventions were designed and delivered by professionals with little input from target populations (2). A rapid evidence review of community engagement approaches found positive impacts on health behaviors, health consequences, self-efficacy and perceived social support (3). Participants engaged in community activities reported physical health benefits such as improved fitness and nutrition though a minority perceived engagement as a drain on energy, time and financial resources (3). Individuals tended to regard community engagement negatively if consultation as opposed to empowerment was the main purpose of professional intervention (3).

A further rapid review found that engagement positively impacted on community empowerment in capacity building, skills, knowledge, and developing a united voice (4). Engagement, however, may inadvertently benefit the less disadvantaged groups more than those in greatest need (4). A systematic review of community engagement to reduce health inequalities identified three theoretical models: patient/consumer involvement, peer/lay-developed interventions, and community empowerment (5). Another systematic review of outcomes from engaging communities in health promotion identified effects on health behaviors, health literacy, health service access, and public health planning (6). Components directly affecting health outcomes included intervention delivery, bi-directional learning, collaborative partnerships, genuine power-sharing, and incorporating community voice (6).

The shift to community engagement is underpinned by two theoretical frameworks: community-based participatory research (CBPR) (7) and community-partnered participatory research (CPPR) (8). CBPR equitably involves community members, researchers, and other stakeholders throughout the research process, recognizing unique strengths and combining knowledge with action to improve health outcomes. CPPR extends this practice by emphasizing the equal value of partners in jointly developing, implementing, and disseminating research. CBPR originated in social psychology (6) and is deeply intertwined with the social justice-oriented threads of key proponent Orlando Fals Borda where research was transformed from an extractive academic exercise into a tool for empowerment and social change (9). CBPR is commonly used in public health, medicine, and nursing (7), as it combines knowledge with action to improve community health (10).

In parallel with community engagement, cross-sector collaboration and co-production gained traction as a means of creating equitable partnerships between professionals and communities crucial to improving public services (11). Five co-production principles were identified: building and maintaining relationships, sharing power, reciprocity, valuing all knowledge, and ensuring mutual benefit (12). Essential to co-production is the notion that people using services are hidden resources, not “drains on the system” [(11) p. 11]. Co-production of health care services can lead to better service innovation, cost savings, enhanced patient satisfaction, and increased health outcomes (13). Difficulties in achieving these aims result from professional resistance, patient disengagement and perceived asymmetry of information (13).

A further systematic review of international studies explored co-approaches (co-production, co-design, and co-creation) for working with communities (14). Thematic synthesis (15) generated descriptive themes of bringing everyone together as equal partners, valuing all knowledge, and creative approaches to solve problems (14). Processes involved creating trust and confidence, developing a shared understanding, giving everyone a voice and sense of ownership, and meeting needs. The authors found, however, a lack of robust evaluation and limited evidence of impact on the management of health conditions (14). A scoping review of the influence of co-productive activities on older adult health and wellbeing located three factors: social and physical activities, development of age-friendly environments, and discussions of healthy and active aging (16). Co-creation was facilitated by agreed aims, empowerment, flexibility, recognition of participants’ time, and an accessible location but impeded by lack of time to build trust, low recruitment levels, and paucity of resources.

### 1.1 Background

The current research reports a thematic synthesis of four studies from the first two phases of the three-phase UK-wide interdisciplinary, multicentered “Mobilizing Community Assets to Tackle Health Inequalities” program. Aiming to address neighborhood health inequalities utilizing local, cultural, and natural assets, projects funded under the program involved mental and community health, and creative participatory interventions in rural, heritage, cultural, green and blue spaces (Supplementary Table 1). The synthesis builds on key outcomes from phases one and two, outlined below. The theoretical frameworks that follow were used to interpret the findings from these studies and act as a basis for theoretical interpretation of the current synthesis.

Phase one investigated how community partnerships and cultural and natural assets improved mental and physical health outcomes. Successes of partnership working included being part of a research project for those not normally involved in research, developing established relationships, employing practice- and arts-based methods, sharing funding democratically, and the role of local assets in involving communities (17). Challenges concerned short-term funding and lack of sustainable financial support for redressing the balance of power in favor of communities (17). The most challenging relationships were those with already overburdened health care staff (17). Improvement, innovation, and creativity were vital to service improvement though community partners drew attention to the need for less bureaucracy and complexity with contracts, collaboration agreements and payment (18). Phase two established cross-sectoral consortia and highlighted the advantages of involving a range of people and organizations in university, community and healthcare collaborations for tackling neighborhood health inequalities (19). The authors supported the need for longer-term funding to alleviate the precariousness of fixed-term posts and advocated co-location of services to promote integration and communication. They recommended that community, voluntary and third sector providers should form consortia, functioning in collaboration not competition for funding, thereby providing a single-entry point for decision-makers and commissioners to access multiple services (19). Co-design, co-production and participatory methods were central to strategies involving communities, however developing partnerships and maintaining relationships took time, particularly to establish trust (20).

Phase one analysis drew upon Alliance Theory where a lack of capacity of one partner is offset by the capacity of another, and the strength of partnership is greater than for partners alone (21). Alliance Theory is a variation of Resource Dependency Theory (22), where organizations lacking potential resources seek out compensatory partnerships. Phase two analysis was based on three consortia-building frameworks: Community-based Consortia Development (CBCD) (23), Organizational Development Empowerment Process (ODEP) (24), and Community-Based Consortia Evaluation (CBCE) (25). For the CBCD, successful negotiation of four stages: assembling, ordering, performing, and ending, is critical to consortium formation (23). The ODEP combines locality development and participatory action research to facilitate individual and system-wide empowerment (24). Building on the CBCD and ODEP, the CBCE provides an integrative framework supporting a participatory research model (25).

The objective of the current research was to analyze and aggregate data from focus groups and case studies conducted by members of the “Mobilizing Community Assets” program national coordinating team with cross-sector collaborative projects comprising academic and non-academic, health and social care, local authority, voluntary and community partners. Given the array of potentially contributory factors across these 27 projects, the research question asked what the core components of cross-sector collaborations and community engagement were for tackling neighborhood health inequalities.

## 2 Methods

### 2.1 Analysis

Thematic synthesis was performed on the lowest level of themes derived from thematic analyses of 10 phase one (1) and 15 phase two focus groups (3), and syntheses of 11 phase one (2) and 12 phase two case studies (4) using Lumivero NVivo 14 software. As a Cochrane recommended method of qualitative evidence synthesis (26), thematic synthesis is frequently used in health research (27–29). It is an iterative process that aggregates the data, develops new codes, and descriptive and higher order analytical themes to generate interpretative constructs (15). To ensure methodological rigor and minimize bias, the synthesis was conducted independently by two researchers employing a hybrid coding approach: inductive coding allowed new insights to emerge without influence from pre-conceived categories while deductive coding incorporated existing themes from the four studies for structure. Following an iterative process, researchers moved between coding, reading, and drafting to translate similar concepts into shared themes. Data analysis was grounded in an interpretivist perspective focused on subjective experiences, acknowledging that reality is socially constructed rather than objective. With this approach in mind, researchers jointly discussed the derivation of new themes, particularly the higher order analytical themes, which they reviewed for coherence and accuracy of data representation.

### 2.2 Participants

Participants consisted of voluntary response samples of adults 18 years and above from research teams awarded UKRI funding for 12 months under phase one (January–December 2022) and 9 months under phase two (November 2022–July 2023) of the Mobilizing Community Assets program. Participants comprised 234 from phases one and two focus groups, and 23 from phases one and two case studies completed on behalf of 12,162 participants. (Table 1).

**Table 1:** Focus group and case study participants for phases one and two.

Phase	Research method (No.)	Participants			
		No.	Mean	Median	Range
One	Focus groups (10)	90	9.00	8.50	7 (13–6)
	Case studies (11)	7,149	649.91	235.50	2,754 (2779–25)
Two	Focus groups (15)	144	9.60	6.00	24 (25–1)
	Case studies (12)	5,013	417.75	286.50	2,457 (2465–8)

### 2.3 Materials

Materials comprised the participant information sheet; consent form; privacy notice; eight focus group questions for each phase (Table 2) and a 12-question practice-based case study questionnaire for both phases (Table 3).

**Table 2:** Focus group questions for phases one and two.

Question number	Phase one questions
1.	Can you tell us about the successes and achievements you have had in partnership working and the enablers and opportunities that have led to these achievements?
2.	Can you tell us about the challenges/barriers/limitations you have encountered in partnership working? What are the barriers to achieving your organization’s goals?
3.	We are interested in sustainability. How sustainable do you think the health and wellbeing work of your organization is? *(For example, in terms of funding, longevity of job posts, relationships with partners?)*
4.	Integrated care systems (ICSs) are seeking to achieve integration. What is your view on this please? *(For example, do you have any thoughts on the integration of health and social care with community providers?)*
5.	What does the term ‘health inequalities’ mean to you?
6.	How do you identify and reach individuals/communities from the poorest backgrounds, living in the most deprived areas?
7.	What are some of the opportunities for connecting with those people experiencing the worst inequalities?
8.	What are some of the challenges for connecting with those people experiencing the worst inequalities?

	Phase two questions
1.	Can you tell us about (a) the successes and achievements and (b) any challenges or barriers you have had in building/setting up your phase two consortium?
2.	How have you integrated community researchers/people with lived experience into your project? How has it affected your approach to research? What have you learned from it? What are your key findings?
3.	To what extent do you think your consortium is (i) sustainable and (ii) scalable?
4.	How well do you think your consortium is integrating with existing integrated care systems (ICSs)? Does your consortium provide one-door access for the ICS?
5.	What do you think are the drivers of health inequalities across your local communities?
6.	How is your project addressing health inequalities? What types of information, data and methods are you using to understand inequality?
7.	What do you see as (a) the opportunities and (b) the challenges for connecting with communities experiencing the worst health inequalities?
8.	How do you think the lived experience of individuals can be better integrated into health systems research?

**Table 3:** Case study questionnaire for both phases one and two.

Question number	**Phase one and phase two questions**
1.	Overview: Name of your organization. Add any links to your website or social media. Your name and role. What is the title of the project. Summarize the case study in no more than three sentences.
2.	Setting: Give a brief description of the area where the project occurred, and the organization/s involved in running the project.
3.	Purpose of the project: What was the purpose of the project? What was the challenge or problem that the project has tried to address? What are the stated aims, goals and objectives of the project?
4.	Description of the project: Briefly describe what the project is and/or what it does. When did the project begin and when did it/will it end? Where did it take place? Who was involved in the project?
5.	Methodology: Why was this approach taken? Why was the project set up this way? Did you draw on any evidence or theory-of-change when setting up the project? What other reasons did you have for designing and researching the project in this way?
6.	Participants: Who took part? Indicate the number of people who took part in the project and any demographic information on participants (i.e., gender, ethnicity, age, disability). Describe how they came to join the project.
7.	Data collection: How was data collected for this case study? What research methods were used? What data was collected and by whom (i.e., academics, community researchers, project partners)?
8.	Project impact and outcomes: Has anything changed as a result of the project? What impact has the project had on participants, the wider community, and your organization? Measurable impact recorded; list of outcomes
9.	Enablers and barriers: What factors have supported the project and any positive outcomes? What factors have prevented the project from being more successful?
10.	Key learning: What is the most important thing you learned from this project? What key advice can you give to others starting a similar project?
11.	Next steps and sustainability: How sustainable is the project? Could the project continue? What are the plans for the project in the future and what is needed for this to happen?
12.	Further information: Include any titles/links to further supporting material about the project, e.g., website or evaluation report.

### 2.4 Procedure

Ethical approval was obtained for the research. Principal investigators were informed in their award letters that they would be required to participate in data collection methods coordinated by the university research team supporting the program. As projects finished at different dates due to no-cost extensions, phase one focus groups were held over 16 weeks (November 2022–March 2023); phase two focus groups were held over 31 weeks (October 2023–May 2024); phase one case studies were collected over 31 weeks (June 2023–January 2024); and phase two case studies were collected over 18 weeks (September 2023–January 2024). Focus group questions were emailed 2 weeks in advance and recapped in the focus groups. Verbal consent was obtained to record audio. Online focus groups of between one and 2 h (mean = 97.21, median = 106 min) were conducted in Microsoft Teams with each project. Case study questionnaires distributed in Microsoft Forms took between under 10 min and over 10 h (mean = 157.50, median = 79.50 min) with some completed in several attempts. Findings from focus group analyses, case study syntheses and data obtained from the studies’ authors formed the dataset for the thematic synthesis.

## 3 Results

**Figure 1:**
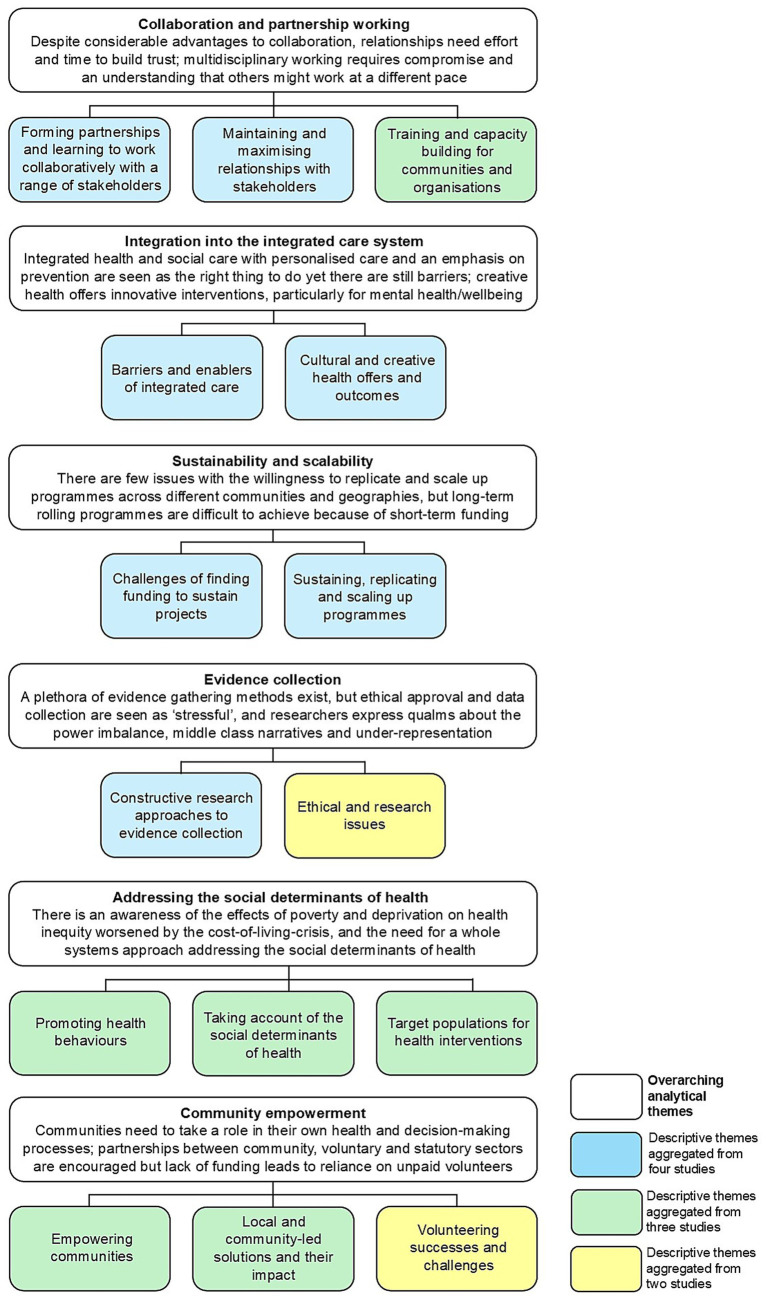
New descriptive themes and overarching analytic themes.

Similarities were sought among the themes from the four studies. Similar themes were included in the synthesis if they occurred twice or more whereas themes with only a single occurrence were excluded as these tended to be context-specific and less transferable to other settings. Similar themes were aggregated to form 15 new descriptive themes. Descriptive themes were tabulated in order of the most studies involved (4, 3 or 2) and the most themes aggregated (range = 7–37, mean = 16.53, median = 15) (Table 4). Seven descriptive themes were derived from four studies, six from three studies and two from two studies. Overarching analytical themes were derived from the new descriptive themes (Figure 1).

**Table 4:** New descriptive themes aggregated from similar themes across studies.

New descriptive themes (no. studies involved/no. themes aggregated)	Focus group themes		Case study themes	
	Phase one	Phase two	Phase one	Phase two
Constructive research approaches to evidence collection (4/37)	Arts-based methods Bespoke assessment tools Case studies Collective evidence Community organizing model Creating new role of research partnership coordinator Index of Multiple Deprivation Joint Strategic Needs Assessment Filmmaking NHS Core20PLUS5 Practice-based evidence Surveys Working with an artist	Asset mapping Co-production Community/ peer researchers Evaluation and outputs Lived experience in research Research methods Working with young people	Mixed methods research User centered and participatory action research frequently used	Building an evidence base for interventions Conducting research, evaluation, and generating data Evidence collection and research to inform policy and practice Impact of initiatives on health outcomes and community wellbeing Understanding the drivers of health inequity to develop targeted interventions Making and extending relationships with community members at the heart of generating data and evidence
Forming partnerships and learning to work collaboratively with a range of stakeholders (4/21)	Building on established programs Collaboration helped by strong existing relationships Collaboration hampered by poor communication Different partnerships at different stages of the project Finding the right partners Getting health professionals on board Understandings between partners	Bringing different people or groups together Collaboration Community organizations Setting up consortia	Collaboration needs to be forced / mediated / find middle ground Ups and downs of multidisciplinary working in academia Finding compromise between different sectors Means of joining up a fragmented ecosystem Multidisciplinary teams as both barriers and enablers	Building consortia and project activities on existing community relationships Collaboration and partnerships as both barriers and enablers Collaboration with diverse stakeholders Different paces and styles of work in different sectors Partnerships between voluntary, community, and statutory organizations providing cohesive support
Barriers and enablers of Integrated care (4/19)	Better organizations to commission services Crisis care Loss of autonomy Lack of communication and consultation One size health and social care will not fit all Piecemeal approaches Poor integration of services Reorganization of integrated care needed Value placed on novelty over efficiency Integrated care systems as the right thing to do	Health and social care Integrated care Local authorities	Good NHS stories Positive outcomes can come from the ability to be flexible with bureaucracy Issues with NHS culture and structure were not conducive to change though when a gap was identified, it was easy to fill it with something new NHS is great when it works but if not there is a bottleneck Referrals and GP knowledge of social prescribing problematic	Emphasis on the integration of health and social care systems to address health disparities
Maintaining and maximizing relationships with stakeholders (4/18)	Fewer long-term relationships Links lost through COVID-19 Need health service input Need supportive partners Not enough time to establish long-term relationships Relationship building Relationships with individuals not organizations	Funding and payment Importance of relationships Importance of timing in relationships	Pace of work and work cultures different across third sector, health and academia Relationship building takes time and effort to build trust and gain local knowledge	Fostering trusted relationships over time Leveraging collective expertise and resources Making and extending relationships with community members is at the heart of generating data and evidence Maintaining contact with groups after projects finish Placing relationships at the center of engaging with communities Supporting relationships in communities
Challenges of finding funding to sustain projects (4/15)	Charging for sessions Demand for services Economies of scale need sustained funding Lack of funding continuity No longitudinal elements Rigidity of funders Rolling programs Short-term projects having longer-term implications	Further funding bids Lack of sustainable financial support	Funding as both a barrier and an enabler	Short-term funding streams. Finding sources of ongoing funding Repositioning funding to respond to existing and emerging community health needs especially post project Things can slow down or halt if further funding is not in place
Cultural and creative health offers and outcomes (4/14)	Alleviating visits to accident and emergency departments Co-design of services Opportunities of collaboration with creative practitioners	Creative health and social care Mental health	Improvement, innovation and development of current services Making things less bureaucratic and less complex can have an impact Psychological impact: wellbeing, confidence, learning, attachment Real systemic change Showing others nationally and internationally how to solve a common systemic problem	Arts-based interventions Creative and cultural engagement Developing sustainable models for integrating community assets into health systems Focus on local community and cultural assets for health
Sustaining, replicating and scaling up programs (4/14)	Challenges in building capacity Consistency of research staff Continuing the intervention Difficulties of setting up programs from scratch Eco-friendly projects and biodiversity action plans Fewer people running programs	Reference to the community ecosystem Scalability Sustainability	Understanding scalability	Creating frameworks and infrastructures that can be scaled up and replicated in other regions Developing sustainable models for integrating community assets into health systems Maximizing impact and on a broader scale Scaling successful models and replicating interventions across different communities
Empowering communities (3/19)	Building relationships of trust Changing behaviors Collective approach to redressing the balance of power Community involvement Community mistrust of authority Community groups without websites Local, informal and not-for-profit groups Negative perception of coproduction Place-based factors Resilient young people Trust in community organizations		Ethnic minority and low take up of activity Low income and incentivization	Collective enthusiasm recognized as a significant input that helped motivate work based in communities Co-production of services Empowering communities to take an active role in own health and wellbeing Engaging community members in decision-making processes Community-based activities require coordination of multiple people, timelines and organisations, adding complexity Putting people first through interactive and co-produced activities
Local and community-led solutions and their impact (3/16)	After-school clubs Engaging with the museum Help to establish community interest companies Impact of the arts Novel ways of offering support Overheads of local organizations Wider role of museums		Can be initiated by faith leaders Give flexibility to the third sector and it can prosper Working with policymakers and stakeholders to create supportive environments for community health initiatives. Influencing policy and system change by advocating integration of community-led approaches into broader health and social care	Demonstrating impact of initiatives on health outcomes and community wellbeing Fostering community-led solutions Involving community members and people with lived experience Mapping existing community assets Utilizing participatory methods to ensure that services are tailored to needs and preferences of the community
Taking account of the social determinants of health (3/14)	Broader structural systemic issues Cost-of-living crisis Disproportionate effect of health inequalities Effects of poverty Highest levels of need and lowest levels of uptake Poor accommodation Poverty and deprivation Socio-economic context	Addressing health inequalities Using inequalities data Overall drivers of health inequalities Whole systems approach needed to tackle health inequalities		Tackling health inequalities by addressing social determinants of health Addressing specific health disparities, such as breast cancer screening uptake in deprived areas
Target populations for health interventions (3/13)	Children not engaging with nature Forced labor Holiday hunger program Interventions with schools Migrants People not engaged with medical services People with learning disabilities Tackling inequalities early	Housing and homelessness		Aiming to provide equitable access to healthcare and support services, particularly in disadvantaged communities Building and evidencing community asset partnerships in housing and health Reaching people who might benefit but are reluctant to participate Addressing gaps in services particularly in rural locations
Training and capacity building for communities and organizations (3/11)	Continuing professional development Different disciplines in the team Instilling best practice Learning from each other Offering training to volunteers Stakeholder advisory groups and events	Importance of training and capacity building		Capacity building within communities and organisations Training community members, peer researchers and stakeholders to sustain and expand their impact Links and trust building help delivery of learning activities Knowledge produced through activities deepens community engagement
Promoting health behaviors (3/10)	Engaging with people and their stories Getting the message out there Networks in place Recruiting young advisors Swimming for long-term health conditions Touch-screen tablet loan	Connecting with communities Integrating people with lived experience		Encouraging health-promoting behaviors Enhancing access to healthcare and support services
Ethical and research issues (2/20)	Disappointing outcomes Ensuring research is useful Evidencing importance of museums Lack of research progress Measuring outcomes Middle class narratives Moral dilemma as researchers Not enough time for participatory action research Obtaining NHS / ethical approval Obtaining research evidence Obtaining the right type of research evidence Power imbalance Stress of gathering evidence Trying to triangulate different methods	Both accessible and inaccessible assets Ensuring all voices are heard in a multi-disciplinary approach, especially under-represented voices Feeling that community/peer researchers are less capable than university researchers and that quantitative methods carry more weight than qualitative methods Need more focused research on how and why to employ a consortium approach Redressing the balance of power in favor of communities Resistance to experts by experience		
Volunteering, successes and challenges (2/7)	Limited capacity of schools to support volunteers Recruitment and safeguarding Reduction in the number of volunteers Volunteering is assumed to be free labor Volunteers paying for own debarring service clearance			Encouraging partnerships between voluntary, community, and statutory organizations to provide cohesive support Funding shortages lead to community organizations relying on volunteers to staff their projects

Colors in Table 3 align with those in Figure 1.

### 3.1 New analytical and descriptive themes

#### 3.1.1 Collaboration and partnership working

Collaboration and partnership working was not always easy but worked better when strong relationships were formed and stakeholders fostered an understanding of different people from different sectors wanting to work in different ways (19), as a university researcher noted: “we had various levels of existing connection with NHS and third sector providers… the ease of navigating these systems… was dependent on the strength of the relationships we were able to develop” [(18) p. 21]. Stakeholders, similarly, commented “having a core of really strong existing relationships” which provided “the central scaffolding on which to bolt or attach any other partnerships was really helpful… with each of us bringing in a different network of partners” [(17) p. 9].

#### 3.1.2 Integration into the integrated care system (ICS)

Integrated health and social care, with a focus on personalized and preventive approaches, was regarded as preferred practice however persistent barriers indicated that no single model was universally applicable. A university researcher proposed “ICS is obviously the right thing to do and a more integrated approach to commissioning and delivery of services across the whole of health and social care is a good idea and an unsolved problem that’s been around since 1948” [(17) p. 11]. Community members were critical of integrated care, saying it was not creating the space to effectively mobilize assets (19) and they needed to have “holistic wellbeing, not crisis care” [(17) p. 11]. Creative health interventions were seen as providing adaptable and innovative solutions particularly for mental health and wellbeing; a practitioner noted that “third sector organizations were better able to respond flexibly and agilely to the development of a new model of working” [(18) p. 21].

#### 3.1.3 Sustainability and scalability

Willingness to replicate and scale up programs across diverse communities and other regions was high, as a principal investigator explained “you would probably have differences across geographies, which is one of the things we were we were going to test… to do things at scale” (19). Scaling up, however, was dependent on obtaining further and longer-term funding, as a principal investigator reflected “it just creates the cycle of chasing the money” [(17) p. 11]. The impact of short-term funding led another investigator to state “it’s something that haunts us, the precariousness of research contracts for early career researchers… practitioners who are delivering are as equally subject to precarious employment conditions” [(17) p. 11]. As most funding was for the research element of a project not delivery, researchers identified the importance of “ways to make the money flow from a funding bid through to real grassroots organizations” (19).

#### 3.1.4 Evidence collection

Evidence collection and evaluation methods included arts-based, participatory and ethnographic research. The process of ‘proving’ that the program worked, however, was seen as stressful and sometimes pointless. Arts-based methods were popular among community researchers attempting to communicate complex ideas, saying “art unlocked a whole other way of doing that” [(17) p. 6]. Another researcher expressed “it’s the richness of having things like photographs or arts-based work where people are telling stories through images or sculptures” [(17) p. 9]. A community organization member expressed that “to be part of a project where we are actually evaluating it and systematically trying to research it… that’s a really positive thing” [(17) p. 6]. Conversely, a third sector representative explained that evaluation was “a huge source of stress” for their practitioners concerned about the quality of the offer being “reduced in favor of quantity” and the “focus on statistics in terms of people through the door rather than the effects afterwards” [(17) p. 10]. University researchers expressed qualms about conducting research within communities, particularly with people working voluntarily, saying “there’s been a total power imbalance… why should anyone agree to give up their time if they aren’t going to be properly rewarded for it?” [(17) p. 15].

#### 3.1.5 Addressing the social determinants of health

Against a backdrop of poverty and deprivation, worsened by the cost-of-living crisis and the need for a whole systems approach, projects acknowledged that engaging with communities to promote health behaviors could alleviate health inequity. A project in the North West of England used storytelling to promote the uptake of screening after exploring different avenues for amplifying health messaging (19). A health practitioner expressed: “you do not start with what’s problematic with their individual behavior but… the socioeconomic context in which they exist that then informs the types of health issues they experience” [(17) p. 12]. A creative practitioner explained that “if families are living in difficult circumstances, then their access to enrichment and therapeutic opportunities is hugely limited” [(17) p. 12]. Researchers were concerned they were unaware of those in most need, questioning how they “reach the folk who we might classify as hard to reach who we know are those who would benefit most?,” highlighting the paradox that “the highest levels of need are often accompanied by the lowest levels of uptake” [(17) p. 13].

#### 3.1.6 Community empowerment

Projects acknowledged that to address health inequity, communities should be empowered to take a role in their own health and decision-making, but this needed to be community led and incorporate lived experience. A university researcher explained the importance of ensuring that “community services are perceived as co-produced with the community and undertaken for the community, rather than there being external agencies providing services to fix the community” [(17) p. 14]. A community researcher expressed a shared view that it was “the system that’s driving under-representation of people to feel totally disengaged and powerless, it’s the system that has to change, not the people” [(17) p. 6]. Projects appreciated the impact of local solutions; a co-investigator explained that people “trust the organizations on their doorstep” [(17) p. 14] Another university researcher described how short-term projects deterred volunteers because of the “loss of legacy for these programs and for the voluntary groups involved” [(17) p. 11].

## 4 Discussion

The aim of the research was to determine the core components of cross-sector collaborations for tackling health inequalities. Thematic synthesis of focus group and case study evidence found that core components were successful collaboration and partnership working; integration into local ICSs and community ecosystems; building sustainable and scalable models of working; trialing evaluation methods; being aware of social determinants; and empowering communities to take a role in their own health and decision-making processes.

Previous research highlighted the importance of collaboration, bi-directional learning and incorporating community beneficiaries into the research process (6). The current study found that projects involving community members in intervention delivery were perceived to have trust and credibility, aligning with the peer/lay intervention model (5); peers shared lived experience, culture, or language which in turn increased uptake and cultural relevance to give a better understanding of local norms and increase the acceptability of interventions (5). Involving peers provided benefits for recipients while empowering lay deliverers with increased skills and confidence (5). Co-creation, however, was hindered by a lack of time to build trust, paucity of resources or structure (16) or uncertainty about how communities could be engaged (5). Projects involved in the current study engaged communities by using combined arts and counselling, culture- and nature-based activities, storytelling and walking, with varying degrees of success, though their remit was not to ascertain if specific ways of engaging communities were better than others.

Core components for tackling health inequalities included integrated care, seen as the right course of action even though variation in individual needs with one size health and social care not fitting all, was identified. A rapid evidence review of enablers and barriers of integrated care illustrated similarly that key enablers included positive attitudes towards the concept of integration (30). The review authors found that employing a person-centered ethos was an important enabler of integrated care (30), contrary to current finding that addressing differences in individual needs was a barrier. Barriers and enablers, however, may be two poles on the same continuum as once a barrier is surmounted it may become an enabler (30). For instance, when projects overcame the bureaucratic constraints of incorporating arts-based therapies into the NHS, they found that the co-delivery process ran smoothly (18). Further core health offers included innovative community health solutions, in keeping with other studies using creative approaches to solve problems (14). Creative research approaches, such as arts-based and participatory methods, were also useful in evaluating the efficacy of projects. However, elements of the evaluation process such as ethical approval, data collection and the academic nature of evaluation were stressful and associated with concerns about power imbalances, middle class narratives, and under-representation.

Components additionally fundamental in tackling health inequalities addressed power imbalances in communities, impacts of local and community-led solutions, and volunteering. Community engagement was empowering at personal and community levels despite empowerment varying in meaning across studies (4). As with the current study, authors found that community empowerment was linked to enhancing mutual support and collective action to mobilize resources (5). Components indirectly affecting health outcomes included building social capital and community capacity, and empowerment of community members (6). A review showed that community empowerment occurred through enhancing mutual support and collective action to organize resources (5) and, partly true here, the lack of sustainable funding to community organizations for service delivery was the main reason why communities felt unempowered. Volunteering was important in some projects for tackling health inequalities because of benefits such as social inclusion, forming networks, new knowledge, confidence, and feeling valued (31). A review found a fifth of studies suggested that promoting community engagement enabled voluntary groups to forge better links with the wider community (4). The current study, however, found increased reliance of community organizations on unpaid volunteers due to the lack of sustainable funding to pay employees.

Replicating and scaling up longer-term programs across different communities in the face of short-term funding were important factors in addressing neighborhood health inequalities. A Canadian report identified three types of scaling: scaling out, scaling up, and scaling deep (32). Scaling out referred to impacting greater numbers by geographic replication whereas scaling up involved impacting law and policy” (32). Scaling deep was related to impacting cultural roots, spreading ideas and using stories to shift norms and beliefs (32). For the current study, reference to scaling projects generally implied scaling out, with some projects incorporating policy changes for scaling up. In the long-term, scaling deep is required to achieve cultural shifts necessary to fully address neighborhood health inequalities, particularly in relation to accessing underserved communities.

Other themes for tackling neighborhood health inequalities concerned the awareness of poverty and deprivation, taking account of social determinants of health and promoting health behaviors. Contrary to positive outcomes from employing a social determinants approach (4, 33), such as the adoption of holistic biopsychosocial interventions, a review identified issues including low perceived self-efficacy of health care workers, lack of training and role modelling, and absence of communities of practice (33). Evidence from a review identifying community engagement approaches suggested that interventions which improved social inequalities also improved health behaviors (3). Conversely, another review found that although community engagement was connected with improved social capital, cohesion, and empowerment among disadvantaged populations, it did not affect mortality, morbidity, or health behaviors (4).

Several theoretical frameworks elucidate the dynamics observed across the program. Alliance Theory provides an understanding how asset sharing and collaboration in phase two created partnerships that strengthened over time (21), for instance, housing providers and health services pooled resources to compensate for gaps in each other’s provision. Findings emphasized the fragility of relationships; partnerships starting to flourish dissolved once funding ended and key personnel were lost, demonstrating that sustained investment is crucial to preserving collective capacity. The CBPR framework that requires a similar alliance between researchers and community members to agree on goals in a collaborative, purposeful partnership was implicitly reflected in projects emphasizing participatory methods, shared decision-making, and co-production (6, 7). It was speculated that co-produced public services and welfare systems would be “more equitable, responsive and creatively designed and delivered” [(11) p. 17] as co-production involving community members in service delivery “shifts the balance of power, responsibility and resources from professionals more to individuals” [(11) p. 11–12].

The CPPR model (8) arguably offers a closer fit in that it explicitly foregrounds power-sharing and co-ownership of the research process. The model was relevant when concerns were raised about under-representation or risk of reproducing deficit narratives; CPPR’s focus on mutual benefit and capacity-building addresses these concerns by prioritizing community-defined assets and outcomes (8). The ODEP (24) further situates partnership work within a broader agenda of system-level change, supporting both individual and organizational empowerment. When combined with the CBCE (25), the approach provides a roadmap for the staged growth of partnerships. Assembling and performing as a consortium over time enabled greater coherent engagement with commissioners and created a single-entry point for local services. Together, these frameworks emphasize that successful collaborations require intentional development, ongoing reflection, and shared accountability to survive beyond initial funding cycles.

### 4.1 Limitations

Limitations include the relatively low number of articles for a thematic synthesis compared to other health-related studies, though a high number of participants with over 400 involved in focus groups and 12,000 in case studies. Furthermore, unlike quantitative research where sample sizes are calculated, thematic synthesis focuses on depth and breadth of the data rather than a specific number of studies. In employing thematic synthesis, the researchers recognize that they were never removed from the research process and acknowledge their understanding of the data may have predisposed them to certain conclusions. Two researchers involved in the current study acknowledge their input in the studies synthesized, though an independent researcher was involved in the reviewing process. Owing to the limitations, broad consensus about conclusions regarding the core components of cross-sector collaborations for tackling neighborhood health inequalities should be treated with relative caution. Certain findings, such as those regarding approaches community engagement and cross sector collaborations are likely to be transferable to other contexts whereas those that are context-dependent, such as with specific populations or in particular settings, are less likely to be transferable.

## 5 Conclusion

The current research conducted a thematic synthesis connecting different sources of information to determine the core components for tackling neighborhood health inequalities. Findings underscore the necessity of building credibility and trust, understanding cultural relevance to address non-participation by underserved communities, and enabling community empowerment. Improving individual and population health requires cross-sector collaboration not just involving communities, universities and healthcare organizations but engaging other sectors, such as training, housing and legal services. Specific aspects of cross-sector collaborations, including sustaining relationships and empowering communities, were associated with successful reduction in neighborhood health inequalities though tended to be cost-, labor-, and time-intensive. Although the projects navigated multiple and compound forms of disadvantage, those which applied an explicit intersectionality lens, such as through listening to the voices of people with lived experience, offered deeper insights as to why varied approaches were necessary across different contexts. Furthermore, community engagement might only improve health among disadvantaged populations if models are designed thoroughly and implemented using effective consultation and participation processes. The thematic synthesis analyzed and aggregated original data from four “Mobilizing Community Assets” studies embodying 27 diverse projects with over 12,000 participants to make an innovative contribution to the body of research concerned with tackling health inequalities. The research provides a more complete knowledge of community engagement and cross-sector collaborations than might have been derived from a single study.

## Data Availability

The raw data supporting the conclusions of this article will be made available by the authors, without undue reservation.

## References

[1] Butterfoss FD Kegler MC. "Toward a comprehensive under-standing of community coalitions: moving from practice to theory". In: DiClemente R Crosby R Kegler MC, editors. Emerging Theories in Health Promotion Practice and Research, 2nd Edn. San Francisco, CA: Jossey-Bass (2002). p. 157–93.

[2] Oliver S Peersman G. Using Research for Effective Health Promotion. Buckingham: Open University Press (2001).

[3] Attree P French B Milton B Povall S Whitehead M Popay J. The experience of community engagement for individuals: a rapid review of evidence. Health Soc Care Community. (2011) 19:25060. doi: 10.1111/j.1365-2524.2010.00976.x 21138495

[4] Popay J Attree P Hornby D Milton B Whitehead M French B . Community Engagement in Initiatives Addressing the wider Social Determinants of Health: A Rapid Review of Evidence on Impact, Experience and Process. Lancaster: University of Lancaster (2007).

[5] O’Mara-Eves A Brunton G McDaid D Oliver S Kavanagh J Jamal F . Community engagement to reduce inequalities in health: a systematic review, meta-analysis and economic analysis. Public Health Res. (2013) 1:40. doi: 10.3310/phr01040 25642563

[6] Cyril S Smith BJ Possamai-Inesedy A Renzaho AM. Exploring the role of community engagement in improving the health of disadvantaged populations: a systematic review. Glob Health Action. (2015) 8:29842. doi: 10.3402/gha.v8.29842, 26689460 PMC4685976

[7] Collins SE Clifasefi SL Stanton J The LEAP Advisory Board Straits KJE Gil-Kashiwabara E . Community-based participatory research (CBPR): towards equitable involvement of community in psychology research. Am Psychol. (2018) 73:884–98. doi: 10.1037/amp0000167, 29355352 PMC6054913

[8] Wells K Jones L. “Research” in community-partnered, participatory research. JAMA J Am Med Assoc. (2009) 302:320–1. doi: 10.1001/jama.2009.1033, 19602693 PMC3050488

[9] Gutiérrez J. Participatory action research (PAR) and the Columbian peasant reserve zones: the legacy of Orlando Fals Borda. (2016). Available online at: https://www.developmenteducationreview.com/issue/issue-22/participatory-action-research-par-and-colombian-peasant-reserve-zones-legacy-orlando (Accessed April 30, 2026).

[10] Wallerstein N Duran B Oetzel J Minkler M. Community-Based Participatory Research for Health: Advancing social and Health Equity. 3rd ed. San Francisco: Jossey-Bass (2017).

[11] Boyle D Harris M. The Challenge of Co-Production: How Equal Partnerships Between Professionals and the Public are Crucial to Improving Public Services. London: NESTA (2009).

[12] National Institute for Health Research. Involve: Guidance on Co-Producing a Research project. London: NIHR (2019).

[13] Palumbo R. Contextualizing co-production of health care: a systematic literature review. Int J Public Sect Manag. (2016) 29:72–90. doi: 10.1108/IJPSM-07-2015-0125

[14] Grindell C Coates E Croot l O’Cathain A. The use of co-production, co-design and co-creation to mobilize knowledge in the management of health conditions: a systematic review. BMC Health Serv Res. (2022) 22:877:1–26. doi: 10.1186/s12913-022-08079-y 35799251 PMC9264579

[15] Thomas J Harden A. Methods for the thematic synthesis of qualitative research in systematic reviews. BMC Med Res Methodol. (2008) 8:45. doi: 10.1186/1471-2288-8-45, 18616818 PMC2478656

[16] Terkelsen AS Wester CT Gulis G Jespersen J Andersen PT. Co-creation and co-production of health promoting activities addressing older people: a scoping review. Int J Environ Res Public Health. (2022) 19:3043. doi: 10.3390/ijerph192013043, 36293629 PMC9602529

[17] Thomson LJ Waterson H Chatterjee HJ. Successes and challenges of partnership working to tackle health inequalities using collaborative approaches to community-based research: mixed methods analysis of focus group evidence. Int J Equity Health. (2024) 23:1–22. doi: 10.1186/s12939-024-02216-1, 38965627 PMC11223342

[18] Mughal R Scherer IA Smithson J Bagnall AM South J Chatterjee HJ. Mobilizing Community Assets to Tackle Health Inequalities: A Case Studies Synthesis and Review. London: University College London (2024).

[19] Thomson LJ Waterson H Chatterjee HJ. Building research consortia to tackle health inequalities: thematic analysis of collaborative approaches for community-led solutions. BMC Public Health. 10.1186/s12939-024-02216-1 PMC11223342 38965627

[20] Mughal R Reynolds R Thomson LJ Waterson H Manley K Gilmore C . Mobilizing Community Assets to Tackle Health Inequalities: A Synthesis and Review of Phase 2 Case Studies. London: University College London (2024).

[21] Iyer E. Theory of alliances. J Nonprofit Public Sect Mark. (2003) 11:41–57. doi: 10.1300/J054v11n

[22] Heide JB. Interorganizational governance in marketing channels. J Mark. (1994) 58:1–15. doi: 10.1177/002224299405800106

[23] Bailey D Koney KM. Developing Community-Based Consortia: An Integrative Framework for Health Social Work Professionals. Report Number WP-92-05. Cleveland: Case Western Reserve University (1992).

[24] Bailey D. Organizational change in a public school system: the synergism of two approaches. Soc Work Educ. (1992) 14:94–105. doi: 10.1093/cs/14.2.94

[25] Bailey D Koney KM. An integrative framework for the evaluation of community-based consortia. Eval Program Plann. (1995) 18:245–52. doi: 10.1016/S0149-7189(95)00019-4

[26] Noyes J Harden A Ames H Booth A Flemming K. Cochrane-Campbell Handbook for Qualitative Evidence Synthesis: Version 1.0. London: Cochrane Publications (2023).

[27] Broad KL Sandhu VK Sunderji N Charach A. Youth experiences of transition from child mental health services to adult mental health services: a qualitative thematic synthesis. BMC Psychiatry. (2017) 17:1–11. doi: 10.1186/s12888-017-1538-1, 29183289 PMC5706294

[28] Fadyl JK Anstiss D Reed K Levack WMM. Living with a long-term health condition and seeking paid work: qualitative systematic review and thematic synthesis. Disabil Rehabil. (2022) 44:2186–96. doi: 10.1080/09638288.2020.1826585, 33016147

[29] Rains LS Echave A Rees J Scott HR Taylor BL Broeckelmann E . Service user experiences of community services for complex emotional needs: a qualitative thematic synthesis. PLoS One. (2021) 16:e0248316. doi: 10.1371/journal.pone.0248316, 33914750 PMC8084224

[30] Thomson LJ Chatterjee HJ. Barriers and enablers of integrated care in the UK: a rapid evidence review of review articles and grey literature 2018–2022. Front Public Health. (2024) 11:128647. doi: 10.3389/fpubh.2023.128647 PMC10794528 38239795

[31] Thomson LJ Elsden E Chatterjee HJ. Volunteering for wellbeing: improving access and social inclusion by increasing the diversity of museum volunteer training for public-facing roles. Mus Soc. (2023) 21:12–30. doi: 10.29311/mas.v21i1.3786

[32] Riddell D Moore M-L. Scaling Out, Scaling Up, Scaling Deep: Advancing Systemic Social Innovation and the Learning Processes to Support it. Montreal: JW McConnell Family Foundation.

[33] Andermann A. The social determinants of health in clinical practice: a framework for health professionals. Can Med Assoc J. (2016) 2016:E474–83. doi: 10.1503/cmaj.160177, 27503870 PMC5135524

