# Supplementary material for: Tackling neighborhood health inequalities in the UK through cross-sector collaborations and community engagement: thematic synthesis of focus group and questionnaire data
Source: Front Public Health. 2026 May 29;14:1832109. doi: 10.3389/fpubh.2026.1832109 (PMC13260637; doi:10.3389/fpubh.2026.1832109)
Supplement: Supplementary file 1 [file Data_Sheet_1.pdf]

Supplementary table 1. Overview of phase one and two Mobilizing Community Assets projects

Phase	Project	Intervention	Location(s)	Lead university
One	Art at the Start	Creative community arts-based intervention for perinatal and infant mental health	Multiple sites across the Scottish Lowlands	University of Dundee
	Arts and Culture in Health Ecosystems (ARCHES)	Exploring how community organizations use arts, culture and nature to improve health via 'community anchors'	Halifax; Bedford; Southwark, London; Birmingham	Leeds Beckett University
	Arts for the Blues	Towards integrating the use of the arts in healthcare and cultural settings to tackle depression and improve wellbeing	Greater Manchester, NW England	Edge Hill University
	Branching Out	Exploring arts-based nature activities to tackle mental health disparities in schools with community 'Artscapers'	Cambridgeshire and Peterborough	UCL/Anglia Ruskin University
	Connecting Roots	Using design to cocreate and support the scaling up of a green social prescribing network for health and wellbeing	Walsall, Birmingham	Royal College of Art
	Phoenix takes Flight	Exploring usability and scalability challenges with community-based health support via social prescribing	Lancashire; South Cumbria	Lancaster University
	Prescribe Heritage Highland	Delivering and scaling up cultural and nature-based non-pharmaceutical interventions in remote and rural areas	Scottish Highlands	University of the Highlands and Islands
	Rooted in Nature	Scaling up nature-based activities for a diverse group of young people, utilizing photo-elicitation, walking interviews, and an embedded researcher model	Middlesbrough	Newcastle University
	Scaling up Human Henge	Using prehistoric cultural heritage sites to enhance mental health well-being in marginalized communities	Wiltshire	Bournemouth University
	Scaling Up Inspiring Ashfield	Extending place-based social prescribing support building on previous community asset mobilization	Ashfield district, Nottinghamshire	Nottingham Trent University
	Social Prescribing for All (SP4ALL)	Increasing diversity in cultural and natural social prescribing programs through shared training	Derby	University of Derby
	Wild Swimming and Blue Spaces	Mobilizing interdisciplinary knowledge and partnerships to combat health disparities at scale	Nottinghamshire	University of Nottingham
Two	Art at the Start	Perinatal arts therapy hub and partnerships (continuing from phase one)	Dundee; Fife; Glasgow; North Uist	University of Dundee
	Building a Well Communities Research Consortium	Developing a collaborative, cross-sectoral and interdisciplinary partnership to address health disparities through integrated care and community assets	East London; Northamptonshire	City, University of London
	Housing-Health Community Asset Partnerships	Building and evidencing community asset partnerships in housing and health to address health disparities	North Cumbria; North East England	Northumbria University
	Building Research by Communities to address Inequities Through Expression (ReCITE)	Using storytelling and other creative methods to improve trust, mental wellbeing, and engagement in services like cancer screening and immunization	Anfield; Everton; Liverpool, Merseyside	Liverpool School of Tropical Medicine
	Challenging Health Outcomes and Integrating Care Environments (CHOICE)	Creative/arts-based and voluntary services support for people living with serious mental illness	Northern Ireland	Ulster University

Common Health Catalyst	Developing a community research consortium to address health disparities by connecting local organizations with health systems to embed co-production	Lanarkshire, Scotland	Glasgow Caledonian University
Creating Change	Collaborative action inquiry approach for integrating creativity and community assets into integrated care system responses to health disparities	West Yorkshire	University of Huddersfield
Devon Community Assets Research Collaborative (CAN-DO)	Aiming to create sustainable partnerships that connect local activities with the integrated care system to improve health outcomes	Devon	University of Plymouth
FLYDE Coast Research Consortium	Improving health and wellbeing on the Fylde Coast by bringing together residents, service providers, local authorities, and academics	Blackpool; Fylde Coast; Lancaster	Lancaster University
INNOVATE	Utilizing community-based research and co-produced, asset-based solutions to enhance mental health equity through co-production	Essex	University of Essex
Living Roots Project	Building a community asset and research consortium to address health equity, focusing on early childhood development	West London	University of Sussex
Organizations of Hope	Building a creative health coalition to address health equity and improve health outcomes through arts and cultural assets	Greater Manchester	University of Manchester
Pathways to Health through Cultures of Neighborhoods	Training young people as researchers and advocates to use cultural engagement to improve young people's health outcomes and life chances	Southampton	University of Southampton
REALITIES in Health Disparities	Using systems thinking to tackle health inequalities by focusing on intergenerational trauma, social injustice, and economic hardship	Clackmannanshire; Easter Ross; North Lanarkshire, Scotland	University of Edinburgh
Social Innovation Coalition for Inclusive Health	Building upon previous research to embed community-driven solutions into public health systems to tackle health disparities through social innovation	East London	University College London
Weston-super-Mare Consortium	Harnessing community assets to tackle inequities and reduce social isolation in end-of-life care and bereavement	Weston-super-Mare, North Somerset	University of Bristol
